# Associations between Gene-Gene Interaction and Overweight/Obesity of 12-Month-Old Chinese Infants

**DOI:** 10.1155/2022/1499454

**Published:** 2022-03-07

**Authors:** Hong Mei, Baoming Yin, Wenhong Yang, Jingli Zhang, Hongyan Lu, Xiaobin Qi, Wenhua Mei, Hongzhong Zhang, Jianduan Zhang

**Affiliations:** ^1^Department of Maternal and Child Health Care, School of Public Health, Tongji Medical College, Huazhong University of Science and Technology, 13 Hangkong Road, Wuhan, Hubei, China; ^2^Wuhan Children's Hospital (Wuhan Maternal and Child Healthcare Hospital), Tongji Medical College, Huazhong University of Science & Technology, 100 Hongkong Road, Wuhan, Hubei, China; ^3^Zhuhai Women and Children's Hospital, 543 Ningxi Road, Zhuhai, Guangdong, China; ^4^Zhuhai People Hospital Affiliated to Jinan University, 79 Kangning Road, Zhuhai, Guangdong, China; ^5^Traditional Chinese Medicine Hospital of Zhuhai in Guangdong Provice, 53 Jingle Road, Zhuhai, Guangdong, China; ^6^Public Hospital Administration of Zhuhai Municipality, 41 Jiaoyu Road, Zhuhai, Guangdong, China

## Abstract

**Background:**

Childhood overweight and obesity (OW/OB) is a worldwide public health problem, and its genetic risks remain unclear.

**Objectives:**

To investigate risks of OW/OB associated with genetic variances in *SEC16B* rs543874 and rs10913469, *BDNF* rs11030104 and rs6265, *NT5C2* rs11191580, *PTBP2* rs11165675, *ADCY9* rs2531995, *FAM120A* rs7869969, *KCNQ1* rs2237892, and *C4orf33* rs2968990 in Chinese infants at 12-month old.

**Methods:**

We conducted a case-control study with 734 infants included at delivery and followed up to 12-month old. The classification and regression tree analysis were used to generate the structure of the gene-gene interactions, while the unconditional multivariate logistic regression models were applied to analyze the single SNP, gene-gene interactions, and cumulative effects of the genotypes on OW/OB, adjusted for potential confounders.

**Results:**

There were 219 (29.84%) OW/OB infants. Rs543874 G allele and rs11030104 AA genotype increased the risk of OW/OB in 12-month-old infants (*P* < 0.05). Those carrying both rs11030104 AA genotype and rs10913469 C allele had 4.3 times greater OW/OB than those carrying rs11030104 G allele, rs11191580 C allele, rs11165675 A allele, and rs543874 AA genotype. Meanwhile, the risk of OW/OB increased with the number of the risk genotypes individuals harbored.

**Conclusions:**

Rs543874, rs11030104, and rs11191580 were associated with OW/OB in 12-month-old Chinese infants, and the three SNPs together with rs10913469 and rs11165675 had a combined effect on OW/OB.

## 1. Introduction

Over the past three decades, the prevalence of childhood overweight and obesity (OW/OB) has dramatically increased worldwide, especially in China [1]. A meta-analysis found that the prevalence of overweight and obesity in Chinese children and adolescents increased from 5.0% and 1.7% in 1991-1995 to 11.7% and 6.8% in 2011-2015, respectively [2]. It is well established that obesity occurred in childhood is a strong predictor of later life obesity and related illnesses such as cardiovascular diseases [3, 4]. The early stage of life, especially the first 1000 days from conception through the age of two years, is increasingly recognized as a critical period for the development of childhood obesity and its adverse consequences [5].

Although social and environmental factors encountered in early life contribute to the development of obesity, the role of genetic influences in the pathogenesis of obesity should not be overlooked [6]. The results from twin studies have reported that around 40-70% of interindividual variability in body mass index (BMI), an indicator commonly used to assess obesity, is attributable to genetic factors [7]. Genome-wide association analysis (GWAS) and meta-analysis have identified more than 100 obesity-related genetic variations in adults [6–8]. However, the found genetic variations could only explain a tiny proportion of obesity heritability (about 2.7% of adult obesity) [9]. The so-called missing heritability may be due to the fact, among many others, that the majority of the investigations have been focusing on a single SNP's effect [10–12], leaving the interactive effect of SNPs overlooked. Some studies have reported that SNPs may influence one another in the development of obesity, generating combined effects. For instance, Liu et al. reported the combinations of *SCAP* rs12487736 G allele, *INSIG2* rs9308762 T allele, and *SREBP2* rs1883205 T allele had an 80% increased risk of obesity compared to those harboring low-risk alleles. At the same time, the single association analyses of the same study only found a significant association between *SCAP* rs12487736 and obesity [13].

Further, the results from studies on genetic variants and obesity have been inconsistent across ages and ethnic groups [14–17]. For instance, Lv et al. reported that among the 19 SNPs previously identified from GWAS of obesity, only *SEC16B* rs543874, *MC4R* rs17782313, *MAP2K5* rs2231323, and *KCTD15* rs11084753 were significantly associated with the risk of obesity in school-aged children [15]. However, the results from Zandona et al. indicated that among the 10 analyzed gene variants, the *BDNF*, *TMEM18*, and *NEGR1* gene variants were associated with BMI *z*-score (BMI_Z), with the *BDNF* variants' effect emerging at the age of one year, and *TMEM18* and *NEGR1* gene variants taking effect at 3.5 years of age [18]. It is unclear whether the gene expressions vary with age, resulting in inconsistency. Furthermore, previous studies have been primarily focused on European ethnic, little is known of the Asian child population [19]. It would be interesting to understand whether the same obesity-associated loci contribute to obesity risk across a range of ancestries or if there are obesity susceptibility genes unique to specific ancestries [20].

As a treasure trove of fundamental insights into the genetic basis of obesity, we reviewed GWAS and meta-analysis studies for BMI in both Asian and European adults and chilren [21]. Finally, based on the selecting strategies (details in Method Strategies for Candidate Gene Selection), we focused on *SEC16B*, *BDNF*, *NT5C2*, *PTBP2*, *ADCY9*, *FAM120A*, *KCNQ1*, and *C4orf33* [8, 22, 23]. The eight genes were reported in GWAS to be associated with obesity in the Asian adults or European children. However, the association between these genes and obesity was seldom reported in Asian children, along with the gene-gene interactions. Therefore, based on the prospective birth cohort study in Zhuhai, China, we set up a case-control study firstly to investigate the impact of genetic variance in SEC16B rs543874 and rs10913469, *BDNF* rs11030104 and rs6265, *NT5C2* rs11191580, *PTBP2* rs11165675, *ADCY9* rs2531995, *FAM120A* rs7869969, *KCNQ1* rs2237892 and *C4orf33* rs2968990 on OW/OB of 12-month-old infants and to examine the interactive and cumulative effects of the risk alleles.

## 2. Materials and Method

### 2.1. Study Population and Design

We conducted a case-control study with 12-month-old infants selected from a prospective birth cohort conducted during 2014 and 2017 in Zhuhai, China [23, 24]. The inclusion criteria of 12-month-old infant participants were: (1) single born with no obvious congenital diseases, had Apgar scores ≥7 in both one and five minutes immediately after birth; (2) had umbilical cord blood collected at birth; (3) followed up at 1, 3, and 12-month old. Infants were divided into OW/OB and normal-weight groups according to their BMI, calculated as body weight (kg)/length(m) [2]. Signed informed consent was obtained from parents of the eligible infants prior to the recruitment. In addition, ethical approval was obtained from the Ethics Committees of the Tongji Medical College, Huazhong University of Science and Technology (Ethical approval number: IORG0003571).

### 2.2. Data Collection

Information on some critical variables that are commonly reported to be associated with childhood obesity, such as maternal prepregnancy and paternal height and weight, age, educational level, monthly household income, and smoking behavior prior to pregnancy [24, 25], was collected at recruitment using a structured questionnaire. Information on maternal gestational weight gain, gestational week, delivery mode, and child sex were obtained from hospital medical records. Birth weight and length were collected at delivery by centrally trained nurses. Umbilical cord blood was collected at delivery by trained nurses, centrifugated, and stored at -80°C within two hours after being collected. Infants' weight and length at the 1-, 3-, and 12-month-old were measured by trained research assistants in the study hospitals, and feeding practice was collected during face-to-face follow-ups at infants' one-month old.

### 2.3. Strategies for Candidate Gene Selection

The screening strategies used to identify candidate SNPs began with a search of genetic variants related to obesity of the Asian adults or European children identified by GWAS from 2013 to 2016 in Web of Science and PubMed, resulting in a pool of 77 candidate SNPs. Next, as previous studies demonstrated rare genotypes are more likely to result in spurious findings, we included SNPs with MAF > 10% in the HapMap CHB (Han Chinese in Beijing) population (http://hapmap.ncbi.nlm.nih.gov/) [26, 27]. We then tested their linkage disequilibrium (LD) using the Haploview v4.2 software. The tag SNPs were all included in the analysis. One was randomly retained if multiple nontag SNPs were found in the same gene but with strong LD (*r*^2^ ≥ 0.80). The call rate of each SNP was over 95%. This process resulted in a list of 10 SNPs for investigation: *SEC16B* rs543874, and rs10913469, *BDNF* rs11030104, and rs6265, *NT5C2* rs11191580, *PTBP2* rs11165673, *ADCY9* rs2531995, *FAM120A* rs7869969, *KCNQ1* rs2237892, and *C4orf33* rs2968990.

### 2.4. SNP Data Generating

Genomic DNA was extracted from umbilical cord blood samples using a Bioteke DNA Investigator Kit AU18016 (Bioteke, Beijing, China). DNA concentration and optical density were determined using a NanoDrop 1000 spectrophotometer (Thermo Fisher Scientific, Waltham, Massachusetts, USA). According to the manufacturer's protocol, genotyping was performed at BIO MIAO BIOLOGICAL Corporation (Beijing, China) with Sequenom MassArraY platform (San Diego, USA).

### 2.5. Outcome Variables

The outcome variable is infants' weight status at 12-month old, categorized as OW/OB and normal weight using BMI standards. BMI ≥ 85^th^ percentile was set as the OW/OB (case group), and BMI<85^th^ percentile was the normal weight (control group) [28].

### 2.6. Covariables

Maternal age, prepregnancy, weight and height, gestational week, gestational weight gain and paternal weight, and height were continuous variables. Maternal prepregnancy and paternal BMIs were calculated by prepregnancy weight/height [2]. Parental educational levels were categorized as middle school or lower, high school/technical, college/university, and master's degree or advanced. Parental smoking status before pregnancy was dichotomized as yes or no. Monthly household income was grouped as <5001, 5001~8000, 8001~15000, and >15000 RMB. The delivery mode was either virginal delivery or caesarean section.

Infant sex includes boy and girl. Infants' BMI_Z and weight for age *z*-score (WFA_Z) at birth and three months of age were generated according to the WHO Child Growth Standards 2006 [29]. Infants' weight gain from birth to three months was calculated using WFA_Z at 3-month-old subtract WFA_Z at birth. The feeding pattern in the 1-month-old was categorized as exclusive breastfeeding, mixed feeding, and formula feeding. Exclusive breastfeeding was defined as feeding only breastmilk without any other food, including water; mixed feeding was feeding with breastmilk and other food; formula feeding was feeding without any breastmilk [30].

### 2.7. Statistical Analysis

The differences in the distribution of demographic characteristics and genotype frequencies between cases and controls were examined. Category variables were presented as frequency and percentage, with Pearson's chi-square test for two categorical variables and linear-by-linear regression analysis for variables more than two categories. Normally distributed variables were presented as mean and standard deviation, and skewed variables were described as median and interquartile range. *T*-test and Wilcoxon rank-sum test were used to compare the two-group differences for normally distributed variables and skew ones, respectively. The Hardy-Weinberg equilibrium (HWE) for genotypes was assessed by a goodness-of-fit chi-square test among controls.

The association between each SNP of neonates and the risk of OW/OB at 12-month old was evaluated by the odds ratio (*OR*) and its 95% confidence interval (*CI*) using unconditional multivariate logistic regression models, with the adjustment for infants' sex, BMI_Z at birth, feeding pattern at 1-month old, weight gain from birth to 3-month old, maternal prepregnancy, BMI and gestational weight gain, and paternal BMI. In order to increase the statistical power, the most likely inheritance model for each SNP was adopted rather than investigating all the models (dominant, recessive, and additive models) simultaneously. The statistical power was calculated by Power v3.0 to determine the effectiveness of SNPs.

The classification and regression tree (CART) analysis was performed to detect the potential gene-gene interactions [31]. It was constructed by splitting data recursively into binary subsamples, beginning with the root node, including all samples. The Gini criteria were used for a high degree of homogeneity in the terminal nodes or subgroups before growing a tree; after the tree was developed, a pruning procedure was performed to avoid model overfitting. The optimal tree was selected based on the lowest misclassification error rate and can be assessed based on cross-validation. Subgroups of 12-month-old infants with differential risk association of OW/OB were generated according to the terminal nodes of the SNPs belonging to, which meant the gene-gene interaction tree was developed, the interactions and cumulative effects of multiple risk SNPs on infants' OW/OB were examined using unconditional multivariable logistic regression models. According to the number of confounders included in the models, three unconditional regression models were employed: Model 1 is the unadjusted model; Model 2 is the Model 1 adjusted for child sex, first-month feeding pattern, and weight gain from birth to 3-month old; Model 3 is the Model 2 further adjusted for BMI_Z at birth, gestational weight gain, and parental BMI.

All statistical analyses were carried out using SPSS v18.0 (SPSS, Chicago, Illinois, USA), and all *P* values were two-tailed with a statistically significant level set as 0.05.

## 3. Results

### 3.1. Basic Characteristics of Participants in the Case and Control Groups

A total of 734 infants were included in this study. The case and control groups, respectively, contain 219 and 515 infants, with boys accounting for 42% and 55% each. BMI_Z at birth was around -0.5 in both groups. The exclusive breastfeeding rate was 39.7% in the case group and 46.6% in the control group. Compared to the control group, infants in the case group had a significantly greater BMI_Z at 12-month old (1.64 V.S. 0.03) and weight gain from 1- to 3-month old (0.63 V.S. 0.45). Around 70% of the parents were college-educated. Mothers had higher prepregnancy BMI (20.45 V.S. 20.00) and lower prepregnancy smoking rate (0.92% V.S. 3.73%) in the case group than in the control group. The average paternal BMI, gestational weight gain, and gestational week were similar in both groups, i.e., 23 kg/m^2^, 14 kg, and 39 weeks, respectively. Monthly family incomes in the two groups were also identical. The details are illustrated in Table 1.

### 3.2. Characteristics of the 10 Candidate SNPs

Information of gene locations, minor allele frequency, calling rate, and function of the 10 SNPs was shown in Table S1. The MAF for the case and control was similar to those in the HapMap database of CHB. The genotypes for all SNPs in the control group were confirmed to HWE (*P* > 0.05). Genotype frequencies of the 10 SNPs by the group were compared in Table S2. The results from Table S2 showed a statistically significant difference in the genotype of rs543874 and rs11030104 between case and control groups (*P* < 0.05).

### 3.3. The Association between each SNP and OW/OB in 12-Month-Old Infants

As shown in Table 2, *SEC16B* rs543874 and *BDNF* rs11030104 were significantly associated with OW/OB at 12-month old. With confounders adjusted, they were associated with increased risk of overweight/obesity in the dominant and recessive models, respectively (*P* < 0.05). For example, in Model 3, the adjusted OR and 95% CI for rs543874 and rs11030104 were 1.58 (1.11, 2.24) and 1.48 (1.03, 2.12), respectively. No significant association was found between the other 8 SNPs and OW/OB.

### 3.4. The Gene-Gene Interaction Tree Development

**Figure 1:**
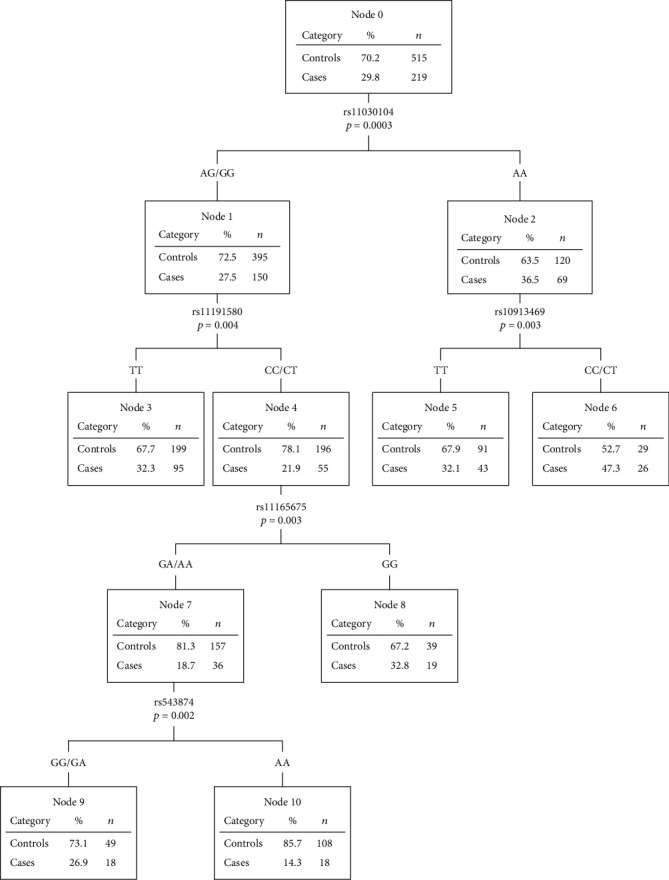
The gene-gene classification and regression tree development of the 10 SNPs.

The CART was depicted in Figure 1. The first split was created by *BDNF* rs11030104 (Node 0), suggesting that *BDNF* rs11030104 posted the highest risk on OW/OB in 12-month-old infants among the 10 SNPs examined. Next, the tree progressed on both daughter nodes (Nodes 1 and 2), and the algorithm created the second split on *NT5C2* rs11191580 and *SEC16B* rs10913469, respectively, resulting in four daughter nodes (Nodes 3, 4, 5, and 6). The next progression was on Node 4, split by *PTBP2* rs11165675, resulting in two daughter nodes (Nodes 7 and 8). After that, *SEC16B* rs543874 split Node 7 into Nodes 9 and 10. Thus, the four levels' gene-gene interaction tree was developed.

### 3.5. Effects of Gene-Gene Interaction on OW/OB in 12-Month-Old Infants

Six gene-gene interaction subtrees were developed by the CART analysis. Among them, the subtree with individuals carrying the combination of *BDNF* rs11030104 G allele, *NT5C2* rs11191580 C allele, *PTBP2* rs11165675 A allele, and *SEC16B* rs543874 AA genotype had the lowest risk for OW/OB in 12-month-old infants. Thus, this terminal node (Node 10) was considered as the reference group in the association analyses. The results from Table 3 showed that except for individuals at the terminal Node 9, individuals at the other four-terminal nodes had a higher risk for OW/OB at 12-month old compared to Node 10 (*P* < 0.05), with the confounders adjusted. For instance, individuals with the combination of *BDNF* rs11030104 AA genotype and rs10913469 CC or CT genotype exhibited the highest risk for OW/OB at 12-month-old infants (OR = 5.31, 95% CI = 2.52 − 11.20) in contrast with the reference group.

### 3.6. The Cumulative Effect of Risk Genes on OW/OB in 12-Month-Old Infants

Cumulative effect analysis of the five risk SNPs identified in the CART analysis was evaluated by unconditional logistic regression analysis. High-risk genotypes were set as *BDNF* rs11030104 AA genotype, *NT5C2* rs11191580 TT genotype, *PTBP2* rs11165675 GG genotype, *SEC16B* rs10913469 CC or CT genotype, and *SEC16B* rs543874 GG or GA genotype according to the results from the single gene association and CART analysis. Participants were categorized into four groups based on the number of risk genotypes they harbored, i.e., 0, 1, 2, and 3-5. The subgroup with 0 risk allele was regarded as the reference group in the analysis. As very few individuals had 4 or 5 risk genotypes (6 cases and 11 controls with 4 risk genotypes; and 1 case and 3 controls with 5 risk genotypes), they were merged with those harboring 3 risk genotypes to form the final subgroup of 3-5 risk genotypes. As shown in Table 4, the risk of OW/OB at 12-month old increased with the number of risk genotypes individuals harbored. For ones with at least 3 risk genotypes, the risk for OW/OB at 12-month old was 3.33 times higher than those without any risk genotype (OR = 4.30, 95% CI = 2.07 − 8.96).

## 4. Discussion

Our study was among the first to evaluate the association between genetic variances and OW/OB of 12-month-old infants in a Chinese population. We found that risk alleles of *SEC16B* rs543874, *BDNF* rs11030104, and *NT5C2* rs11191580 were associated with OW/OB in Chinese infants at the age of 12 months, and *SEC16B* rs543874 and rs10913469, *BDNF* rs11030104, *NT5C2* rs11191580, and *PTBP2* rs11165675 had a combined effect on OW/OB in 12-month-old Chinese infants.

*SEC16B*, a mammalian homolog of *SEC16*, plays a vital role in forming coat protein II vesicles, which mediate protein transport from the endoplasmic reticulum to the Golgi apparatus [32]. It has been reported that obesity may be triggered by the expression of *SEC16B*, which affects the synthesis and transport of lipase, then inhibiting the decomposition of fat [15]. Our results showed that *SEC16B* rs543874 was associated with OW/OB in 12-month-old infants, consistent with a recent report on children aged 5-13 years old in Northern Mexican [33]. Furthermore, it has been reported that rs543874 decreases the binding of SOX6, which was considered a transcription factor contributing to the developmental origins of obesity by promoting adipogenesis [34]. Regarding the association between *SEC16B* rs10913469 and OW/OB in 12-month-old infants, we did not find significant evidence and previous studies also reported conflicting results. For example, a study conducted by Xi et al. showed no association between *SEC16B* rs10913469 and central obesity in 6–18-year-old children in Beijing, China [35]; however, a meta-analysis found that rs10913469 in *SEC16B* gene were significantly associated with the risk of obesity [36]. The inconsistency may, in part, indicate that the expression of genetic variants changes across the life course [37].

*BDNF* is a neurotrophin that plays a fundamental role in the development and plasticity of the central nervous system [38] and was recognized as a major participant in the regulation of food intake and satiety responsiveness [14, 39], locomotor activity [40], and energy metabolism [41]. In our study, a significant association was found between *BDNF* rs11030104 and OW/OB in 12-month-old infants, consistent with the results from a prospective cohort study on four-year-old children [14]. However, we did not find a significant impact of *BDNF* rs6265 on infants' OW/OB at 12-month old. This result is inconsistent with that from a study on school-aged children, where adolescents with *BDNF* rs6265 GG had lower BMI_Z and postprandial glucose levels than those with *BDNF* rs6265 GA/AA alleles [42]. The differences of participants' age and the exclusion of underweight infants in our study might, to some extent, explain the discrepancy of the results.

*NT5C2* encodes a hydrolase that serves an essential role in cellular purine metabolism by acting primarily on inosine 5'-monophosphate and other purine nucleotides [43]. This study detected significant association between rs11191580 and OW/OB in 12-month-old Chinese infants. The impact of *NT5C2* rs11191580 polymorphism on infants' obesity may function through the region of linkage disequilibrium of several genes (*NT5C2*, *CYP17A1*, and *CNNM2*). *CYP17A1* gene expression alters the biosynthesis of steroid hormones, which was reported to reduce adiposity in Japanese women [44]. *CNNM2* (ancient conserved domain protein, *ACDP2*) is a transporter of magnesium, which is required for the catalytic activity of numerous metalloenzymes [45]. Thus, those genes may promote body fat changes by regulating body cell synthesis.

The *PTBP2* rs11165675 allele identified by GWAS in European adults failed to confer susceptibility to obesity in our study [9]. However, our results agreed with the findings of GWAS from Felix et al. in European children [8]. The protein encoded by *PTBP2* binds to intronic polypyrimidine clusters in pre-mRNA molecules and is implicated in controlling the assembly of other splicing-regulatory proteins [46]. However, the mechanism of *PTBP2* regulating obesity remains unrevealed.

*ADCY9* plays a role in G protein-coupled receptors, *FAM120A* participates in RNA binding, *KCNQ1 is* involved in a potassium channel that plays an essential role in a number of tissues, and *C4orf33* works on protein binding. Although the common variances in *ADCY9* rs2531995, *FAM120A* rs7869969, *KCNQ1* rs2237892, and *C4orf33* rs2968990 were reported to be associated with childhood or adulthood obesity in GWAS or meta-analysis studies [8, 9], we failed to identify any apparent association in 12-month-old infants. This inconsistency might indicate that the effects of variance in *ADCY9* rs2531995, *FAM120A* rs7869969, *KCNQ1* rs2237892, and *C4orf33* rs2968990 may function differently across age and ethnics. Further investigations are warranted to clarify our results.

It has been estimated that genetic factors contribute to 20-60% of interindividual variability in BMI [7] and more than 100 genes were found to be related to obesity. However, the heritability analysis of the obesity-related genes could explain very little of BMI variation [9]. The potential mechanism underlying this missing heritability might include the complex interplay among genes [13]. In this study, cumulative analysis of rs10913469 (effective allele: CC/CT), rs543874 (effective allele: GG/GA), rs11030104 (effective allele: AA), rs11191580 (effective allele: TT), and rs11165675 (effective allele: GG) showed a strong combined effect on OW/OB susceptibility in 12-month-old infants, demonstrating that genes may interact each other to promote the pathogenesis or progression of OW/OB in infants.

Apart from the genetic effects, we also found that weight gain velocity from birth to 3 months old had an impact on OW/OB in 12-month-old infants (Table S3), which was consistent with previous studies on weight gain velocity and obesity [47, 48]. However, infant sex, first-month feeding pattern, BMI_Z at birth, parental BMI, and gestational weight gain showed insignificant association with OW/OB in our study. Our previous cohort study found that maternal prepregnancy obesity, excessive gestational weight gain, and feeding pattern during the first 6 months of life were correlated with rapid weight gain during infancy [49]. Thus, based on our previous findings and the current results, we hypothesize that compared to the influence of parental factors, infant feeding pattern and BMI_Z at birth, and infant weight gain velocity might have a more direct and substantial impact on infant obesity.

To the best of our knowledge, this is the first try to explore the genetic susceptibility of OW/OB on gene-gene interaction networks in children at the early years of life. In this study, we found three SNPs associated with OW/OB in infancy, and genes could function interactively on the development of obesity. However, there are several limitations in our study. Firstly, as the study participants are one-year-old infants, the obesity rate was low. Therefore, we used the BMI percentiles for overweight and obesity classification and merged overweight and obesity to reach a comparable rate of obesity between cases and controls. Moreover, the classification of feeding patterns in our study was based on the first-month breastfeeding situation. As most children had complementary feeding practice after six months of age, the feeding frequency and quantity could be considered. In our study, we considered if there was breastfeeding at six and 12 months old with no significant difference between the case and control groups (data not shown) but did not include the time, frequency, and quantity of complementary food introduction, which made it one of the limitations in our study. More research in a large, diverse population is needed to clarify our findings and explore the underneath mechanisms of the connections.

## 5. Conclusions

In summary, using the data from the prospective birth cohort, we demonstrated for the first time that SEC16B rs574367 and rs543874, BDNF rs11030104 and NT5C2 rs11191580 had an independent effect on infants' OW/OB in 12-month-old Chinese infants; the gene-gene interaction effects of them (adding PTBP2 rs11165675) were also detected, together with a substantial cumulative effect. More studies are needed to confirm our findings in different population and to explore the mechanisms of the interactive and cumulative effects between genes.

## Figures and Tables

**Table 1:** General characteristics of participants in case and control groups.

	Case group (*N* = 219)	Control group (*N* = 515)	*P*
Infants			
Boys, *N* (%)	92 (42.01)	284 (55.15)	**0.0011**
BMI_Z at birth	-0.52 (1.18)	-0.46 (1.15)	0.5528
BMI_Z at 12-month old	1.64 (1.31, 2.00)	0.03 (-0.53, 0.50)	**<.0001**
Feeding pattern in the 1-month old, *N* (%)			0.1330
Exclusive breastfeeding	87 (39.73)	240 (46.60)	
Mixed feeding	106 (48.40)	232 (45.05)	
Formula feeding	26 (11.87)	43 (8.35)	
Weight gain velocity (0-3 months old)	0.63 (-0.07, 1.56)	0.45 (-0.36, 1.18)	**0.0028**
Mothers			
Age (year)	28.82 (26.97, 31.47)	28.44 (26.50, 31.00)	0.1430
Educational level, *N* (%)			0.6459
Middle school or under	10 (5.46)	32 (7.24)	
High school/technical	33 (18.03)	82 (18.55)	
University/college	128 (69.95)	290 (65.61)	
Master's degree or advanced	12 (6.56)	38 (8.60)	
Prepregnancy smoking, *N* (%)	2 (0.92)	19 (3.73)	**0.0378**
Prepregnancy BMI (kg/m^2^)	20.45 (18.82, 22.55)	20.00 (18.25, 21.99)	**0.0209**
Gestational weight gain (kg)	14.10 (11.76, 17.00)	14.40 (12.00, 17.00)	0.7177
Gestational week (week)	39.29 (38.57, 40.00)	39.29 (38.43, 40.14)	0.8229
Caesarean section, *N* (%)	85 (38.81)	184 (35.73)	0.4275
Fathers			
Educational level, *N* (%)			0.5527
Middle school or under	11 (6.04)	24 (5.52)	
High school/technical	21 (11.54)	68 (15.63)	
University/college	133 (73.08)	310 (71.26)	
Master's degree or advanced	17 (9.34)	33 (7.59)	
BMI (kg/m^2^)	23.66 (21.72, 26.23)	23.82 (21.63, 25.65)	0.6166
Smoking, *N* (%)	57 (27.54)	143 (28.83)	0.7288
Household monthly income (yuan/RMB), *N* (%)			0.9618
<5001	98 (44.75)	197 (38.25)	
5001~8000	26 (11.87)	126 (24.47)	
8001~15000	66 (30.14)	128 (24.85)	
>15000	29 (13.24)	64 (12.43)	

Note: ^∗^refers to significant difference between the groups, *P* < 0.05; results from variables with normal distribution were presented as mean (standard deviation), with skewed distribution that were present as median (interquartile range), results from category variables were presented as frequency (percentage).

**Table 2:** Associations between each SNP and risk for overweight/obesity at 12-month-old infants.

Characteristics	Model 1 unadjusted analysis		Model 2 adjusted analysis ^a^		Model 3 adjusted analysis ^b^	
	OR (95% CI)	*P*	OR (95% CI)	*P*	OR (95% CI)	*P*
*SEC16B* rs543874		**0.02**		**0.01**		**0.01**
AA	1		1		1	
GG+GA	1.48 (1.05, 2.08)		1.57 (1.11, 2.23)		1.58 (1.11, 2.24)	
*SEC16B* rs10913469		0.09		0.11		0.11
TT	1		1		1	
CC+CT	1.34 (0.96, 1.87)		1.32 (0.94, 1.85)		1.32 (0.94, 1.86)	
*BDNF* rs11030104		**0.02**		**0.03**		**0.03**
AG+GG	1		1		1	
AA	1.53 (1.08, 2.18)		1.50 (1.04, 2.14)		1.48 (1.03, 2.12)	
*BDNF* rs6265		0.05		0.09		0.10
CT+TT	1		1		1	
CC	1.43 (1.00, 2.04)		1.38 (0.96, 2.99)		1.36 (0.94, 1.98)	
*NT5C2* rs11191580		**0.04**		0.09		0.06
TT	1		1		1	
CC+CT	0.71 (0.52, 0.98)		0.75 (0.54, 1.04)		0.73 (0.52, 1.02)	
*PTBP2* rs11165675		0.06		0.11		0.08
GA+AA	1		1		1	
GG	1.43 (0.99, 2.07)		1.36 (0.93, 1.99)		1.40 (0.96, 2.06)	
*ADCY9* rs2531995		0.13		0.12		0.18
TT	1		1		1	
CC+CT	0.68 (0.41, 1.11)		0.67 (0.40, 1.11)		0.70 (0.42, 1.17)	
*FAM120A* rs7869969		0.33		0.43		0.40
AG+GG	1		1		1	
AA	0.84 (0.59, 1.20)		0.87 (0.60, 1.24)		0.86 (0.60, 1.23)	
*KCNQ1* rs2237892		0.23		0.18		0.24
CT+TT	1		1		1	
CC	0.82 (0.59, 1.13)		0.80 (0.57, 1.11)		0.82 (0.58, 1.14)	
*C4orf33* rs2968990		0.18		0.35		0.37
TT	1		1		1	
CC+CT	1.34 (0.88, 2.05)		1.23 (0.80, 1.89)		1.22 (0.79, 1.88)	

Notes: the unconditional multivariable logistic regression models were adopted; ^a^adjusted for child gender, feeding pattern in the 1-month old, weight gain velocity from birth to 3-month old; ^b^adjusted for all variables of Model 2, including BMI_Z at birth, gestational weight gain. and parental BMI.

**Table 3:** Risk estimates of gene-gene interactions on OW/OB in 12-month-old infants.

Terminal node (subtree group)	Genotype	OR (95% CI)	*P*
10	rs11030104 (AG/GG)- rs11191580 (CC/CT)- rs11165675 (GA/AA)- rs543874 (AA)	1	
9	rs11030104 (AG/GG)- rs11191580 (CC/CT)- rs11165675 (GA/AA)- rs543874 (GG/GA)	1.96 (0.90, 4.26)	0.09
8	rs11030104 (AG/GG)- rs11191580 (CC/CT)- rs11165675 (GG)	2.78 (1.29, 5.98)	<0.01
3	rs11030104 (AG/GG)- rs11191580 (TT)	2.74 (1.55, 4.85)	<0.01
6	rs11030104 (AA)- rs10913469 (CC/CT)	5.31 (2.52, 11.20)	<0.01
5	rs11030104 (AA)- rs10913469 (TT)	2.51 (1.33, 4.76)	<0.01

Note: ORs of terminal nodes were calculated by unconditional multivariable logistic regression analysis, adjusting for child gender, BMI_Z at birth, feeding pattern in the 1-month old, weight gain velocity from birth to 3-month old, gestational weight gain, and parental BMI. CART: the classification and regression tree.

**Table 4:** Cumulative effect of the risk SNPs between case and control groups.

Number of risk genotypes	Case group *N* (%)	Control group *N* (%)	OR (95% CI)	*P* ^∗^
0	11 (5.98)	71 (15.96)	1	—
1	59 (32.07)	172 (38.65)	2.15 (1.06, 4.38)	0.60
2	58 (31.52)	119 (26.74)	3.19 (1.55, 6.56)	0.04
≥3	56 (30.43)	83 (18.65)	4.30 (2.07, 8.96)	<0.01
Linear-by-linear regression analysis		*P* ^#^ < 0.01		

Notes: ORs of number of risk genotypes were calculated by unconditional multivariable logistic regression analysis, adjusting for child gender, BMI_Z at birth, feeding pattern in the 1-month old, weight gain velocity from birth to 3-month old, gestational weight gain, and parental BMI;^∗^*P* from unconditional multivariable logistic regression analysis;^#^*P* from linear-by-linear regression anlaysis.

## Data Availability

Data is available to access by email Prof. Jianduan Zhang, jd_zh@hust.edu.cn.

## Supplementary Materials

Supplementary Materials Table S1: the characteristics of the 10 candidate SNPs. Table S2: genotype frequencies of the SNPs in case and control groups. Table S3: risk estimates between covariables and OW/OB in 12-month-old infants.
