# Supplementary material for: Associations between Gene-Gene Interaction and Overweight/Obesity of 12-Month-Old Chinese Infants
Source: Biomed Res Int. 2022 Mar 7;2022:1499454. doi: 10.1155/2022/1499454 (PMC8920651; doi:10.1155/2022/1499454)
Supplement: Supplementary Materials — Table S1: the characteristics of the 10 candidate SNPs. Table S2: genotype frequencies of the SNPs in case and control groups. Table S3: risk estimates between covariables and OW/OB in 12-month-old infants. [file 1499454.f1.docx]

|  | Table S1 Basic characteristics of the 10 SNPs. | | | | | | |  |  |
| --- | --- | --- | --- | --- | --- | --- | --- | --- | --- |
| Gene name | SNP | chromosome | MA^a^ | MAF^b^ | MAF^c^ | MAF ^d^ | Calling rate (%) | SNP function description | Gene function description |
| *SEC16B* | rs543874 | chr1:177889479 | G | 0.20 | 0.20 | 0.18 | 98.9 | Decrease binding of SOX6 | Plays a role in the organization of the endoplasmic reticulum exit sites. |
| *SEC16B* | rs10913469 | chr1:177913518 | C | 0.22 | 0.22 | 0.23 | 98.1 | Intron variant, decrease binding of NCOR2 |  |
| *PTBP2* | rs11165675 | chr1:97039967 | A | 0.50 | 0.50 | 0.47 | 97.5 | Intergenic variant, decrease binding of GATA2 | RNA-binding protein which binds to intronic polypyrimidine tracts and mediates negative regulation of exons splicing. |
| *C4orf33* | rs2968990 | chr4:130879073 | T | 0.38 | 0.40 | 0.44 | 98.9 | Intergenic variant, decrease binding of RORC | Protein binding. |
| *FAM120A* | rs7869969 | chr9:96217446 | G | 0.48 | 0.48 | 0.49 | 97.3 | Intron variant, decrease binding of DNMT1 | RNA binding. |
| *NT5C2* | rs11191580 | chr10:104906210 | C | 0.23 | 0.23 | 0.24 | 99.0 | Intron variant, decrease binding of CUX1 | May have a critical role in the maintenance of a constant composition of intracellular purine/pyrimidine nucleotides in cooperation with other nucleotidases. |
| *BDNF* | rs11030104 | chr11:27684516 | G | 0.45 | 0.45 | 0.40 | 99.2 | Intron variant, decrease binding of BCL11A | During development, promotes the survival and differentiation of selected neuronal populations of the peripheral and central nervous systems. |
| *BDNF* | rs6265 | chr11:27679915 | T | 0.37 | 0.47 | 0.36 | 98.1 | Missense variant, increase binding of MYC |  |
| *KCNQ1* | rs2237892 | chr11:2839750 | T | 0.46 | 0.37 | 0.32 | 98.8 | Intron variant, decrease binding of NR2C2 | Potassium channel that plays an important role in a number of tissues, including heart, inner ear, stomach and colon. |
| *ADCY9* | rs2531995 | chr16:4013466 | T | 0.35 | 0.35 | 0.26 | 100.0 | Intron variant, decrease binding of SETDB1 | Adenylyl cyclase that catalyzes the formation of the signaling molecule cAMP in response to activation of G protein-coupled receptors. |
| Note: ^a^ MA: minor allele; ^b^ MAF: minor allele frequency in the case group; | | | | | | | |  |  |
| ^c^ MAF: minor allele frequency in the control group; | | | | | | | |  |  |
| ^d^ MAF for CHB in the database of HapMap. | | | | | | | |  |  |

| Table S2 Genotype frequencies of the 10 SNPs in the case and control groups | | | | | | |
| --- | --- | --- | --- | --- | --- | --- |
| SNPs | Genotype | Case group [N (%)] | Control group [N (%)] | *P*^a^ | *P*^b^ | *p*^HWE^ |
| *SEC16B* rs543874 | AA | 138（63.89） | 369（72.35） | **0.04** | **0.01** | 0.66 |
|  | GA | 68（31.48） | 129（25.29） |  |  |  |
|  | GG | 10（4.63） | 12（2.35） |  |  |  |
| *SEC16B* rs10913469 | TT | 132（61.40） | 343（68.06） | 0.22 | 0.09 | 0.12 |
|  | CT | 70（32.56） | 137（27.18） |  |  |  |
|  | CC | 13（6.05） | 24（4.76） |  |  |  |
| *BDNF* rs11030104 | GG | 48（22.22） | 136（26.56） | 0.05 | **0.03** | 0.27 |
|  | AG | 99（45.83） | 256（50.00） |  |  |  |
|  | AA | 69（31.94） | 120（23.44） |  |  |  |
| *BDNF* rs6265 | TT | 50（23.70） | 139（27.31） | 0.14 | 0.07 | 0.33 |
|  | CT | 98（46.45） | 253（49.71） |  |  |  |
|  | CC | 63（29.86） | 117（22.99） |  |  |  |
| *NT5C2* rs11191580 | TT | 130（60.19） | 265（51.86） | 0.06 | 0.14 | 0.23 |
|  | CT | 71（32.87） | 216（42.27） |  |  |  |
|  | CC | 15（6.94） | 30（5.87） |  |  |  |
| *PTBP2* rs11165675 | AA | 59（27.83） | 137（27.18） | 0.11 | 0.31 | 0.10 |
|  | GA | 94（44.34） | 260（51.59） |  |  |  |
|  | GG | 59（27.83） | 107（21.23） |  |  |  |
| *ADCY9* rs2531995 | TT | 28（12.96） | 47（9.16） | 0.30 | 0.30 | 0.56 |
|  | CT | 94（43.52） | 234（45.61） |  |  |  |
|  | CC | 94（43.52） | 232（45.22） |  |  |  |
| *FAM120A* rs7869969 | GG | 49（22.79） | 105（21.04） | 0.61 | 0.36 | 0.97 |
|  | AG | 107（49.77） | 239（47.90） |  |  |  |
|  | AA | 59（27.44） | 155（31.06） |  |  |  |
| *KCNQ1* rs2237892 | TT | 27（12.56） | 61（11.96） | 0.48 | 0.32 | 0.55 |
|  | CT | 104（48.37） | 225（44.12） |  |  |  |
|  | CC | 84（39.07） | 224（43.92） |  |  |  |
| *C4orf33* rs2968990 | TT | 34（15.74） | 102（20.04） | 0.38 | 0.21 | 0.08 |
|  | CT | 98（45.37） | 225（44.20） |  |  |  |
|  | CC | 84（38.89） | 182（35.76） |  |  |  |

Note: HWE: the Hardy-Weinberg equilibrium; ^a^ *p* value from *Chis*-square test; ^b^ *p* value from linear-by-linear regression analysis.

| Table S3 Risk estimates between co-variables and OW/OB in 12-month-old infants | | | | | |
| --- | --- | --- | --- | --- | --- |
|  | Gene-gene interaction regression model | |  | Cumulative effect analysis model | |
|  | *OR*（95%CI） | *P* |  | *OR*（95%CI） | *P* |
| Child gender | 1.54 (1.10, 2.18) | **0.01** |  | 1.41 (0.97, 2.03) | 0.07 |
| Feeding patter in the 1 month-old | 0.78 (0.60, 1.01) | 0.06 |  | 0.83 (0.63, 0.09) | 0.18 |
| Weight gain velocity from birth to 3 month-old | 1.29 (1.09, 1.54) | **0.01** |  | 1.31 (1.09, 1.58) | **0.01** |
| Gestational weight gain | 1.0 (0.96, 1.04) | 0.97 |  | 1.01 (0.96, 1.05) | 0.84 |
| Child BMI_Z at birth | 1.18 (0.97, 1.42) | 0.09 |  | 1.19 (0.97, 1.46) | 0.09 |
| Maternal BMI | 1.06 (0.99, 1.13) | 0.10 |  | 1.04 (0.97, 1.12) | 0.24 |
| Paternal BMI | 1.01 (0.95, 1.07) | 0.76 |  | 0.99 (0.94, 1.06) | 0.84 |
